# Multifaceted regulation of the sumoylation of the Sgs1 DNA helicase

**DOI:** 10.1016/j.jbc.2022.102092

**Published:** 2022-05-30

**Authors:** Shibai Li, Ashley Mutchler, Xinji Zhu, Stephen So, John Epps, Danying Guan, Xiaolan Zhao, Xiaoyu Xue

**Affiliations:** 1 Molecular Biology Program, Memorial Sloan Kettering Cancer Center, New York, New York, USA; 2 Materials Science, Engineering, and Commercialization Program, Texas State University, San Marcos, Texas, USA; 3 Department of Chemistry and Biochemistry, Texas State University, San Marcos, Texas, USA

**Keywords:** Sgs1, Sgs1–Top3–Rmi1 complex, sumoylation, Esc2, Holiday junction dissolution, HJ, Holiday junction, HR, homologous recombination, MMS, methyl methanesulfonate, MR, midregion, SIM, SUMO-interaction motif, SLD, SUMO-like domain, STR, Sgs1–Top3–Rmi1

## Abstract

Homologous recombination repairs DNA breaks and sequence gaps *via* the production of joint DNA intermediates such as Holliday junctions. Dissolving Holliday junctions into linear DNA repair products requires the activity of the Sgs1 helicase in yeast and of its homologs in other organisms. Recent studies suggest that the functions of these conserved helicases are regulated by sumoylation; however, the mechanisms that promote their sumoylation are not well understood. Here, we employed *in vitro* sumoylation systems and cellular assays to determine the roles of DNA and the scaffold protein Esc2 in Sgs1 sumoylation. We show that DNA binding enhances Sgs1 sumoylation *in vitro*. In addition, we demonstrate the Esc2’s midregion (MR) with DNA-binding activity is required for Sgs1 sumoylation. Unexpectedly, we found that the sumoylation-promoting effect of Esc2-MR is DNA independent, suggesting a second function for this domain. In agreement with our biochemical data, we found the Esc2-MR domain, like its SUMO E2-binding C-terminal domain characterized in previous studies, is required for proficient sumoylation of Sgs1 and its cofactors, Top3 and Rmi1, in cells. Taken together, these findings provide evidence that while DNA binding enhances Sgs1 sumoylation, Esc2-based stimulation of this modification is mediated by two distinct domains.

The Sgs1 DNA helicase in budding yeast and its homologs in other organisms have multiple genome maintenance functions important for the well-being of the organisms (1). Mutations of the human Sgs1 homolog, BLM, underlie the Bloom syndrome characterized by increased levels of DNA crossovers and other forms of genomic instabilities (2). Sgs1 and BLM limit the levels of DNA crossover partly by dissolving Holliday junctions (HJs) into noncrossover repair products (3). In this process, Sgs1 (or BLM) collaborates with the topoisomerase Top3 and its cofactor Rmi1 (or Rmi1/2) (3). Impaired HJ dissolution due to Sgs1–Top3–Rmi1 (STR) deficiency leads to growth defects or even cell lethality when combined with mutations affecting other HJ removal enzymes, such as the Mus81-Mms4 structure specific nuclease, highlighting the importance of STR-mediated HJ removal (4). STR also contributes to other steps of homologous recombination (HR), such as resection of DNA ends and disassembly of D-loop intermediates (5, 6, 7, 8, 9, 10, 11).

Sgs1 and BLM are known to be subjected to multiple types of posttranslational modifications (12). Recent studies have shown that all three subunits of the STR complex are sumoylated and their sumoylation positively influence HJ removal in cells (13, 14). Mechanistically, Sgs1 sumoylation fosters its association with Top3, which contains SUMO-interaction motifs, and helps to enrich STR at DNA repair foci (14). Similar to Sgs1, BLM sumoylation also promotes its roles in HR repair (15, 16).

STR sumoylation in yeast is stimulated by the SUMO E3 ligase (Mms21) that is a subunit of the Smc5/6 complex (13, 14). Similarly, BLM sumoylation depends on the Mms21 homolog in human cells (17). In yeast, STR sumoylation is additionally promoted by the conserved scaffold protein Esc2 (18). Esc2 contains two SUMO-like domains (SLDs) and only the C-terminal SLD2 binds to the SUMO E2 enzyme (Ubc9) and promotes sumoylation (18). In addition to Esc2, *in vivo* STR sumoylation shows a dependency on HJ structures that are present at low levels in cells (14). This dependency can provide a partial explanation for the small percentage of sumoylated forms of STR detected in cells (13, 14, 19). Currently, it is unclear whether HJ structures directly or indirectly contribute to STR sumoylation. Given that Esc2 contains a midregion (MR) exhibiting HJ-binding activity (20, 21), it is possible that Esc2 engagement with HJ structures may stimulate STR sumoylation.

To better our understanding of how Sgs1 sumoylation is regulated, we seek to define the roles of HJ structures and Esc2 using reconstituted Sgs1 sumoylation systems. We show that HJ-DNA stimulates Sgs1 sumoylation in a basal sumoylation system that contains non–DNA-binding sumoylation machinery, providing evidence that Sgs1 binding to DNA *per se* promotes its sumoylation. The observed DNA-based stimulation of Sgs1 sumoylation is enhanced by the Esc2 protein. In addressing whether DNA binding by Esc2 contributes to Sgs1 sumoylation, we generated an Esc2 variant containing point mutations in its MR (Esc2-MR) that abolished its DNA binding without affecting its SUMO E2 interaction. Using this variant and the MR deletion mutant, we provide evidence that Esc2-MR contributes to Sgs1 sumoylation but through a DNA-independent manner, thus uncovering another role for this domain. Consistent with our *in vitro* data, cellular results provided evidence that Sgs1 function and sumoylation are positively affected by both the Esc2-MR domain and its SLD2. Our data thus define the stimulatory elements that render efficient Sgs1 sumoylation in promoting its functions.

## Results

### HJ-DNA promotes *in vitro* Sgs1 sumoylation

Cellular studies have suggested a dependence of Sgs1 sumoylation on the formation of HJ structures, since removal of homologous recombination factors required for HJ formation diminishes Sgs1 sumoylation (14). One explanation for the DNA dependency of Sgs1 sumoylation is that Sgs1 binding to HJ-DNA *per se* favors its sumoylation. To test this idea, we examined the effects of synthetic HJ-DNA on Sgs1 sumoylation *in vitro*.

We have previously established an *in vitro* sumoylation system that can robustly sumoylate Sgs1 when the purified STR complex is incubated with purified sumoylation enzymes (Fig. S1*A*) (18). As seen for other sumoylation reactions, the addition of SUMO E1, SUMO E2, and SUMO in the presence of ATP are sufficient to support basal level of Sgs1 sumoylation, since the SUMO E2 can transfer SUMO to substrates upon its activation by the SUMO E1 at the consumption of ATP (14, 18, 22). For simplicity, this reaction system is referred to as basal sumoylation reaction hereafter. The addition of the SUMO E3 Mms21 bound to its partner Smc5 into this system can lead to high-level Sgs1 sumoylation (18). We confirmed that Mms21–Smc5 exhibit moderate DNA-binding ability (Fig. S1*B*) (23, 24). In contrast, components of the basal sumoylation system, including the SUMO, SUMO E1, and SUMO E2, do not bind DNA (25). Thus, we used the basal sumoylation system without the DNA-binding E3 to address whether Sgs1 binding to DNA *per se* favors its sumoylation. Since sumoylation leads to upshift of protein bands on gels, the level of Sgs1 sumoylation was quantified as the percentage of Sgs1 signals from the upshifted bands against the total Sgs1 signals detected on the immunoblots.

**Figure 1:**
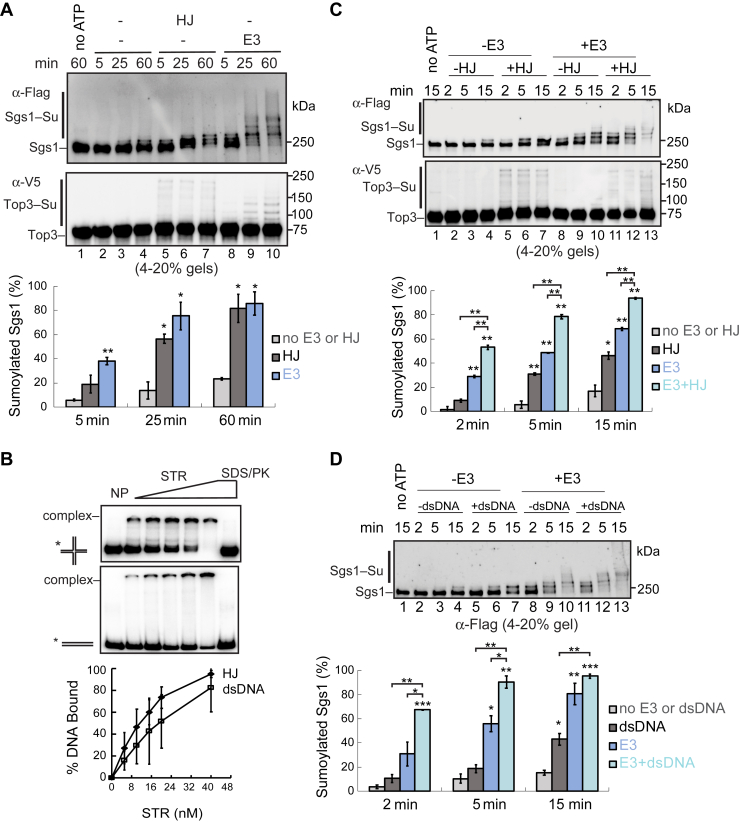
**DNA and the SUMO E3 additively stimulate Sgs1 sumoylation**. *A*, HJ-DNA stimulates Sgs1 sumoylation in the basal sumoylation reactions. The sumoylation assays contains the STR complex, SUMO E1, SUMO E2, SUMO, and ATP in the presence or absence of SUMO E3 and HJ-DNA (see Experimental procedures). Sgs1 was detected *via* the FLAG tag fused to it and Top3 *via* the V5 tag by immunoblotting. The percentage of sumoylated Sgs1 was shown as mean ± SD (n = 2 technical replicates). *B*, DNA mobility shift assay showed that the purified STR complex binds to both HJ-DNA and dsDNA. Interactions between STR and HJ-DNA or dsDNA were indicated by the shifted bands representing DNA–protein complexes. The results were quantified and plotted as mean ± SD (n = 3 technical replicates). *C*, HJ-DNA and the SUMO E3 additively stimulate Sgs1 sumoylation. Sumoylation assays were performed as in panel (*A*), except the SUMO E3 and/or HJ-DNA were added when indicated. The percentage of sumoylated Sgs1 was shown as mean ± SD (n = 2 technical replicates). *D*, dsDNA and the SUMO E3 additively stimulate Sgs1 sumoylation. Sumoylation assays were performed and data presented as in panel (*C*). ∗*p* < 0.05; ∗∗*p* < 0.01; ∗∗∗*p* < 0.001. HJ, Holiday junctions; STR, Sgs1–Top3–Rmi1.

As seen previously, a small percentage of the Sgs1 protein was converted to a monosumoylated form in the basal sumoylation reactions without DNA (Fig. 1*A*, *lanes 1*–*4*) (18). Significantly, the addition of synthetic HJ-DNA led to increased Sgs1 sumoylation and the appearance of multisumoylated or polysumoylated Sgs1 bands (Fig. 1*A*, *lanes 5*–*7*). Quantification showed that at 60 min, a small fraction of Sgs1 (∼20%) was sumoylated without HJ-DNA, while the majority (>80%) of Sgs1 was sumoylated in the presence of HJ-DNA (Fig. 1*A*, *bottom*). Top3 sumoylation showed a similar patten, albeit was inefficient in this setup as seen previously (Fig. 1*A*) (18). Consistent with our previous findings, Rmi1 was not sumoylated in this system, possibly because its sumoylation requires additional factors not present in the assay (18). We confirmed that purified STR complex used here showed robust HJ binding *in vitro* (Fig. 1*B*). Since STR is the only component in the basal sumoylation reaction that binds DNA (25), our data provide evidence that Sgs1 binding to HJ-DNA stimulates its sumoylation. As seen previously, the addition of the Mms21–Smc5 SUMO E3 strongly enhanced the sumoylation of both Sgs1 and Top3 in the absence of DNA (Fig. 1*A*, *lanes 8*–*10*) (18). Given that *in vitro* sumoylation of Sgs1, but not Top3 or Rmi1, was robust, we focused on Sgs1 in subsequent tests.

### DNA and the SUMO E3 additively increase Sgs1 sumoylation *in vitro*

Next, we examined combined effects of HJ-DNA and the Mms21–Smc5 SUMO E3 in Sgs1 sumoylation. We carried out the sumoylation reaction in shorter duration (2–15 min) than those shown in Figure 1*A* (up to 60 min) in anticipation of enhanced STR-sumoylation efficiency with addition of both DNA and the E3. Indeed, at each time point examined, the addition of both HJ-DNA and the SUMO E3 showed stronger stimulation of Sgs1 sumoylation than adding each component alone (Fig. 1*C*). For example, within 5 min of reactions, a majority of Sgs1 (∼80%) was sumoylated in the presence of both HJ-DNA and the E3, compared with approximately 30 to 50% of sumoylated Sgs1 in the presence of either factor (Fig. 1*C*, *bottom*). Thus, HJ-DNA remains stimulatory of Sgs1 sumoylation in the presence of SUMO E3. Previous studies have shown that the Mms21–Smc5 E3 binds to dsDNA (23, 24); we additionally showed that this E3 also bound to HJ-DNA, albeit less pronounced when compared with STR (Figs. 1*B* and S1*B*). It is thus possible that HJ binding by STR and/or the SUMO E3 contributes to the additional stimulation of Sgs1 sumoylation upon the addition of both HJ-DNA and the E3.

HJ-DNA contains four arms of dsDNA and a four-way DNA junction. As STR bound similarly to dsDNA and HJ-DNA (Fig. 1*B*), we asked whether the dsDNA parts of the HJ structure were sufficient to stimulate Sgs1 sumoylation. Indeed, we found that dsDNA promoted Sgs1 sumoylation to a comparable level as HJ-DNA in the basal sumoylation system without the SUMO E3 (Fig. 1, *C* and *D*, *lanes 2*–*4 versus lanes 5*–*7*). For instance, reactions containing either HJ-DNA or dsDNA resulted in about 43% Sgs1 being sumoylated within 15 min (Fig. 1, *C* and *D*, *bottom*). As seen for HJ-DNA, dsDNA also showed additive effects on Sgs1 sumoylation when combined with the Mms21–Smc5 SUMO E3 (Fig. 1, *C* and *D*, *lanes 11*–*13 versus lanes 5*–*10*). For example, Sgs1 sumoylation was seen to reach about 90% within 5 min in the presence of both the SUMO E3 and dsDNA, compared with 18 to 55% sumoylated Sgs1 in the reactions containing either factor (Fig. 1, *C* and *D*, *bottom*). These data provided evidence that STR binding to the dsDNA can facilitate Sgs1 sumoylation, and this effect is augmented by the SUMO E3.

### DNA and the Esc2 protein additively increase Sgs1 sumoylation

**Figure 2:**
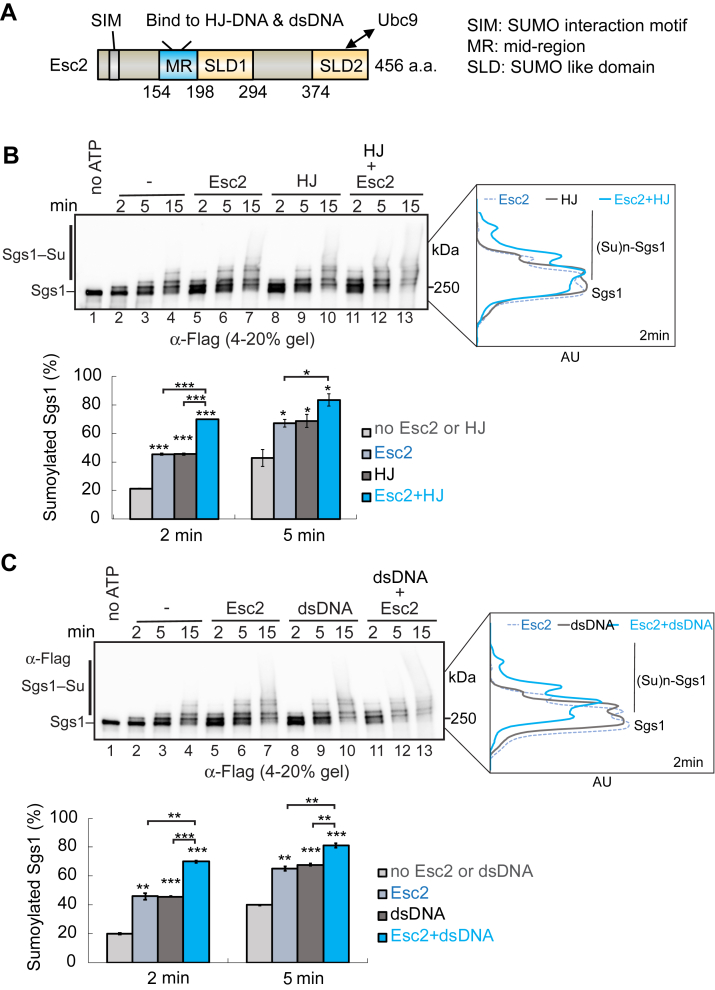
**DNA and Esc2 additively enhance Sgs1 sumoylation**. *A*, schematics of the Esc2 domain structures. *B* and *C*, the addition of Esc2 enhanced the Sgs1 sumoylation in the presence of HJ-DNA (*B*) and dsDNA (*C*). The sumoylation assays were performed similarly as those in Figure 1 with modification as described in the text and Experimental procedures. A representative immunoblotting image was shown. The scans of *lane 5*, *8*, and *11* at 2 min (*right*) highlight the appearance of polysumoylated or multisumoylated Sgs1 forms in Esc2 + DNA conditions. The percentage of sumoylated Sgs1 was shown as mean ± SD (n = 2 technical replicates). ∗*p* < 0.05; ∗∗*p* < 0.01; ∗∗∗*p* < 0.001. HJ, Holiday junction.

An important regulator of the Sgs1 sumoylation is the conserved scaffold Esc2 protein. Esc2 and its homologs contain two SLDs (SLD1 and 2) (Fig. 2*A*) (26). We have previously showed that SLD2, but not SLD1, is required for Esc2-mediated stimulation of Sgs1 sumoylation, and this is achieved *via* SLD2 binding to the backside of the SUMO E2 Ubc9 (18). Other studies have revealed that Esc2 also contains a MR (Esc2-MR, Fig. 2*A*) that exhibits strong preference for binding to HJ-DNA over dsDNA (20, 21). Given that both Esc2 and HJ-DNA promote Sgs1 sumoylation, we examined whether HJ-binding by Esc2 could directly stimulate Sgs1 sumoylation.

To better quantify the influence of Esc2 on Sgs1 sumoylation, higher salt concentration was used to increase the stringency of sumoylation reaction (see Experimental procedures). We first confirmed that Esc2 increased Sgs1 sumoylation in reactions containing SUMO E1, E2, E3, and SUMO (Fig. 2*B*, *lane 5*–*7 versus lanes 2*–*4*) (18). As described previously, HJ-DNA stimulation of Sgs1 sumoylation was seen also in this reaction condition in the absence of Esc2 (Fig. 2*B*, *lanes 8*–*10 versus lanes 2*–*4*). Importantly, we detected additive stimulation of Sgs1 sumoylation by Esc2 and HJ-DNA (Fig. 2*B*, *lanes 11*–*13*). For example, at 2 min reaction time, less than half of Sgs1 (∼45%) was sumoylated in the presence of either Esc2 or HJ-DNA, whereas more than 70% of Sgs1 was sumoylated upon the addition of both (Fig. 2*B*, bottom). Similar observation was made when HJ-DNA was replaced by dsDNA (Fig. 2*C*). It is noteworthy that when Esc2 was included together with either HJ-DNA or dsDNA, the addictiveness in stimulating Sgs1 sumoylation was also evidenced by the increased levels of higher molecular weight Sgs1 bands that represent multisumoylated or polysumoylated Sgs1 (Fig. 2, *B* and *C*, scan at the *right*). These results provided evidence that Esc2 and HJ-DNA or dsDNA additively promote Sgs1 sumoylation.

### Generating Esc2 mutant proteins that abolish its DNA-binding ability

**Figure 3:**
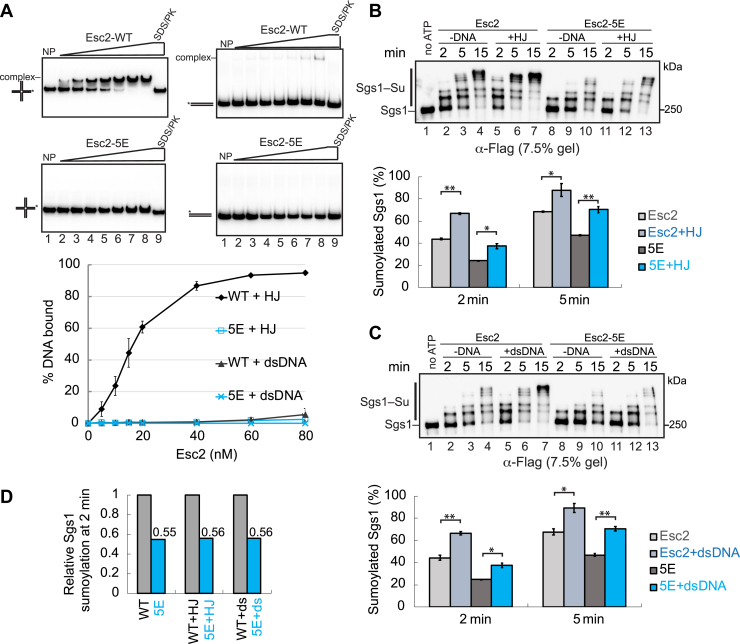
**Esc2-MR mutants reduce Sgs1 sumoylation in the presence or absence of DNA**. *A*, DNA mobility shift assay showed that Esc2 had a strong preference for binding to HJ-DNA compared to dsDNA, while Esc2-5E was defective in binding to either form of DNA. The mean ± SD from at least three independent experiments were plotted. *B* and *C*, Esc2-5E mutant reduced Sgs1 sumoylation levels in the presence or absence of HJ-DNA (*B*) or dsDNA (*C*). Sumoylation assays were performed as in Figure 2. Percentage of sumoylated Sgs1 showed mean ± SD (n = 2 technical replicates). *D*, relative levels of Sgs1 sumoylation was quantified based on data shown in panel (*B*) and (*C*) at 2 min, setting the Sgs1 sumoylation level in reactions containing WT Esc2 as 1.00. ∗*p* < 0.05; ∗∗*p* < 0.01; ∗∗∗*p* < 0.001. HJ, Holiday junction; MR, midregion.

Despite Esc2 has a strong preference of binding to HJ-DNA over dsDNA (Fig. 3*A*) (20), the two forms of DNA exerted similar stimulation of Sgs1 sumoylation in the presence of Esc2 (Fig. 2, *B* and *C*). This result raised the possibility that Esc2-based stimulation of sumoylation may be independent of its DNA-binding ability. To directly test this idea, we first generated an Esc2 variant that abolished its DNA-binding ability. Previous reports have shown that an Esc2-MR spanning its 154 to 198 amino acids is involved in DNA binding (Fig. 2*A*) (20, 21). We confirmed that deleting this region (Esc2-MRΔ) abolished Esc2 binding to either HJ-DNA or dsDNA (Fig. S2*A*). As domain deletion could affect protein folding, we attempted generating point mutations that abolished Esc2 binding to DNA.

Sequence alignment among Esc2 orthologs suggested that several conserved lysine and arginine residues within the Esc2-MR domain could be involved in DNA binding (Fig. S3*A*). We replaced five of these conserved basic residues (K179, K182, K183, K197, and R198) with glutamic acid to generate the Esc2-5E variant (Fig. S3*A*). Both Esc2-5E and the wild-type (WT) Esc2 were purified to homogeneity and showed similar gel filtration elution profiles (Fig. S3, *B* and *C*), suggesting the well-behavior of the mutant protein. Importantly, recombinant Esc2-5E protein abolished Esc2 binding to either HJ-DNA or dsDNA (Fig. 3*A*).

### Esc2-MR promotes Sgs1 sumoylation independently of its DNA-binding ability

Next, we examined Esc2-5E in Sgs1 sumoylation reactions. To better assess the sumoylated forms of Sgs1, which migrated above 250 kD, we used a different percentage of gels than used in the aforementioned figures to enhance the separation of Sgs1 from its sumoylated forms. If Esc2 binding to DNA contributes to Sgs1 sumoylation, we would expect that Esc2-5E reduces Sgs1 sumoylation in the presence of DNA but remains stimulatory in reactions without DNA. However, we found that compared with WT Esc2, Esc2-5E reduced Sgs1 sumoylation in the presence or absence of HJ-DNA (Fig. 3*B*). For example, within 2 min of the reaction, compared with WT Esc2, Esc2-5E led to about twofold reduction of Sgs1 sumoylation with either the presence or absence of HJ-DNA (Fig. 3*B*, *bottom*, Fig. 3*D*). This result provided evidence that the Esc2-MR domain contributes to Sgs1 sumoylation but in a DNA-independent manner. A similar conclusion was reached when examining the reactions containing dsDNA instead of HJ-DNA (Fig. 3, *C* and *D*) or in reactions containing Esc2-MRΔ (Fig. S2*B*). These data strengthened the conclusion that the Esc2-MR domain has a previously unappreciated DNA-independent role in sumoylation. It is likely that the mutated residues in Esc2-5E reduced both DNA binding and sumoylation stimulatory functions.

**Figure 4:**
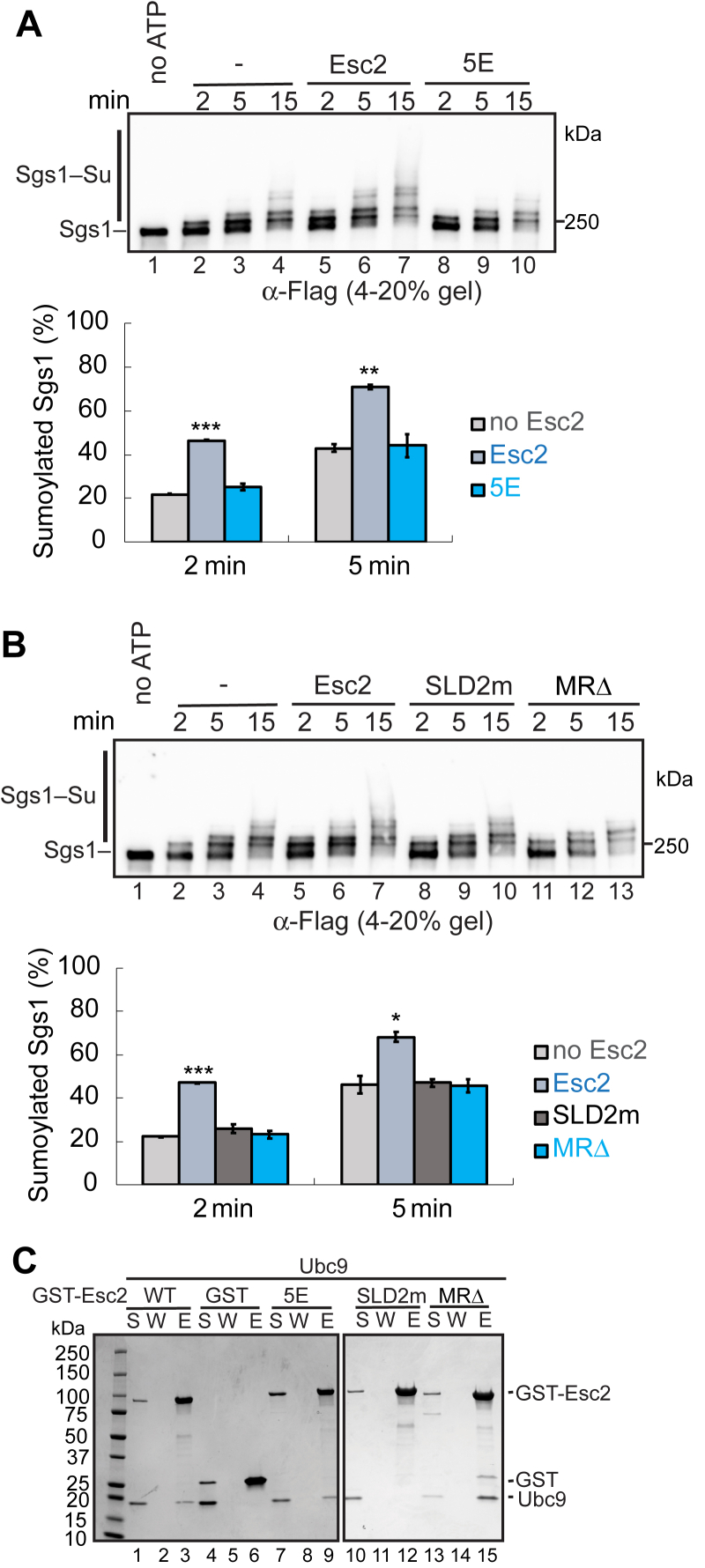
**Esc2-MR contributes to Sgs1 sumoylation independently of Ubc9 binding**. *A* and *B*, Esc2-5E (*A*) and Esc2-MRΔ (*B*) abolished Esc2-mediated stimulation of Sgs1 sumoylation in the absence of DNA, similar to Esc2-SLD2m. Sumoylation assays were performed as in Figure 2, except that no DNA was included. The quantified percentage of sumoylated Sgs1 showed mean ± SD (n = 2 technical replicates). ∗*p* < 0.05; ∗∗*p* < 0.01; ∗∗∗*p* < 0.001. *C*, protein pull-down assay showed that both Esc2-5E and Esc2-MRΔ were proficient for Ubc9 binding *in vitro*. As a control, Esc2-SLD2m abolished Ubc9 interaction. Purified GST, GST-Esc2, or its variant proteins bound to glutathione beads were examined for their abilities to pull down Ubc9. The assay was examined by SDS-PAGE, and pictures of representative gels after Coomassie blue stain are shown. (S) Supernatant, (W) wash, (E) eluate. MR, midregion.

We moved on to assess if the Esc2-MR domain is essential for Esc2-based stimulation of Sgs1 sumoylation. To this end, we compared reactions containing WT Esc2, Esc2-5E, or Esc2-MRΔ with reactions containing no Esc2 in the absence of DNA. We found that the degree of Sgs1 sumoylation was similar amongst the reactions containing Esc2-5E or Esc2-MRΔ *versus* no Esc2 (Fig. 4, *A* and *B*). For example, at the 2 min reaction time point, WT Esc2 renders ∼46% Sgs1 being sumoylated, compared with ∼21% without Esc2 or with Esc2-MR mutant variants (Fig. 4*A*, *bottom*). Further, higher molecular weight sumoylated Sgs1 forms were detected at 5 min time points only in the presence of WT Esc2 but not its MR mutant variants (Fig. 4, *A* and *B*). Collectively, these data provide evidence that Esc2-MR is essential for Esc2-based stimulation of Sgs1 sumoylation in a DNA-independent manner *in vitro*.

### Esc2-MR mutants support Esc2 association with the SUMO E2

We have previously shown that Esc2 uses its SLD2 to associate with the Ubc9 SUMO E2, and this binding is critical for Esc2-based stimulation of Sgs1 sumoylation (18). When compared side by side, the effects of Esc2-MRΔ mutant matched that of the Esc2-SLD2 mutant and both abolished Esc2-based stimulation of Sgs1 sumoylation (Fig. 4*B*). However, unlike Esc2-SLD2m that failed to bind to the Ubc9 SUMO E2 protein, the two Esc2-MR mutant variants maintained this interaction. Specifically, *in vitro* pull-down tests using purified proteins showed that like WT Esc2, Esc2-5E and Esc2-MRΔ mutant proteins associated with Ubc9, whereas the Esc2-SLD2m protein that harbors two mutations (D447A and D449A) in the SLD2 region lost this interaction as seen previously (Fig. 4*C*) (18). These data provide evidence that the detected effects of the Esc2-MR variants are not due to disrupting the Esc2-Ubc9 interaction, though pull-down data could not exclude kinetic alteration of this interaction.

### Esc2-MR contributes to STR sumoylation in cells

**Figure 5:**
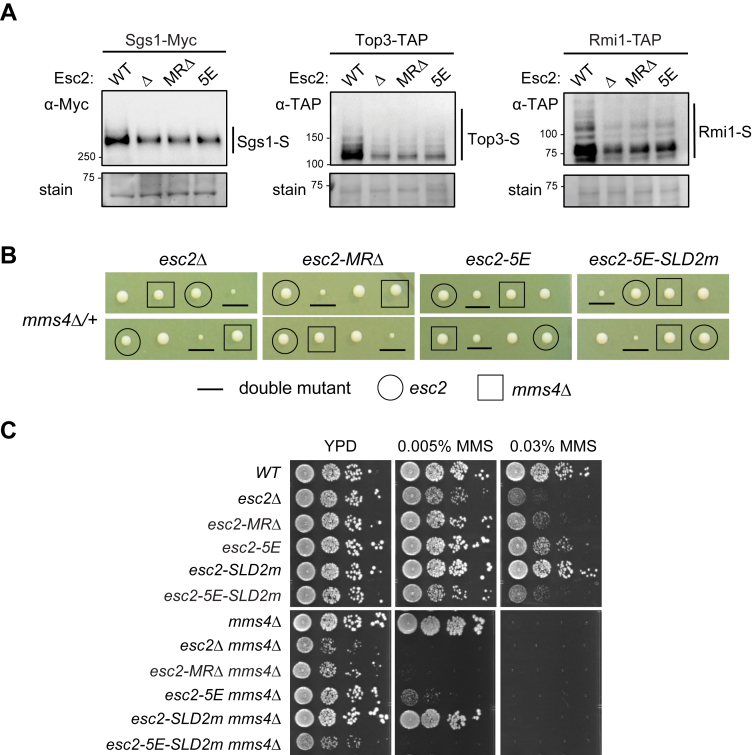
**The Esc2-MR domain aids STR sumoylation**. *A*, sumoylation levels of STR subunits were reduced in *esc2Δ*, *esc2-MRΔ* and *esc2-5E* cells. Sumoylated proteins were enriched due to the binding of 8His-tagged SUMO to the Ni-NTA beads. The elutes from the beads were examined by immunoblotting using the antibodies recognizing the tag fused to the endogenous Sgs1, Top3, or Rmi1, and sumoylated forms of the proteins (-S) are marked. Equal loading is shown by Ponceau S stain (stain). *B*, tetrad analyses from diploid strains with indicated genotypes. Spore clones were grown at 30 °C for 2 days. Spores containing different mutations were identified based on genotyping. Two representative tetrads among at least nine tetrads per diploid strain are shown. *C*, *esc2* mutants worsen the genotoxic sensitivity of *mms4Δ* cells to different degrees. Cells were spotted in 10-fold serial dilutions and grown for 2 days at 30 °C. MR, midregion; STR, Sgs1–Top3–Rmi1.

Next, we used cell-based assays to challenge the conclusion derived from *in vitro* data that Esc2-MR is important for Sgs1 sumoylation. We first addressed how Esc2-MR mutations affect *in vivo* Sgs1 sumoylation. To this end, we replaced WT Esc2 with either the *esc2-5E* or the *esc2-MRΔ* allele at its endogenous locus. Both mutant proteins were expressed close to WT levels, indicating that the examined mutations did not grossly affect protein behavior in cells (Fig. S4). We found that *esc2-5E* or -MRΔ reduced the sumoylation of Sgs1 as well as that of Top3 and Rim1 (Fig. 5*A*). The effects seen for *esc2-5E* and *-MRΔ* was similar to those seen for *esc2Δ* (Fig. 5*A*). Theses data corroborate our *in vitro* findings and support the conclusion that the Esc2-MR domain plays an important role in STR sumoylation.

### Genetic examination of the Esc2-MR alone and in combination with Esc2-SLD2

We then used genetic readouts to assess the effects of *esc2-MR* mutants on HJ removal. The STR-mediated HJ dissolution pathway acts in parallel of the Mus81-Mms4 HJ resolution pathway (4). As such, a genetic readout for impairment in STR-mediated HJ removal can be the sensitization of cells lacking Mms4 (or Mus81), particularly under DNA damage conditions when HJ levels increase (27). If *esc2* mutants diminished STR-based HJ removal, we would expect that they exhibited negative genetic interactions with *mms4Δ*. As reported previously, *esc2Δ mms4Δ* double mutants showed stronger sensitivity toward the DNA methylation agent methyl methanesulfonate (MMS) and slower growth than either single mutant (18) (Fig. 5, *B* and *C*). In addition, the MMS sensitivity of *esc2Δ mms4Δ* was rescued by preventing HJ formation through the removal of the recombinase Rad51 (Fig. S5), indicating that the sensitivity was partly caused by increased HJ levels. Since in *mms4Δ* cells, STR is the main enzyme to remove HJs, this result suggests that *esc2Δ* sensitization of *mms4Δ* is partly due to impairing STR functions.

When *esc2-5E* or -*MRΔ* was combined with *mms4Δ*, the double mutant showed smaller spore clone sizes and increased MMS sensitivity compared with the corresponding single mutants, with a stronger effect seen for *esc2-MRΔ* (Fig. 5, *B* and *C*). In addition, the MMS sensitivity of *esc2-5E mms4Δ* was partially suppressed by *rad51Δ*, as seen for *esc2Δ* (Fig. S5). These results provided genetic evidence that the Esc2-MR domain is involved in STR-based HJ removal, though other interpretations cannot be ruled out.

To test if Esc2-MR may have separate roles from the Esc2-SLD2, we combined mutations of both domains to generate the *esc2-5E-SLD2m* allele, which again supported normal protein levels (Fig. S4). In comparison with *esc2-SLD2m* and *esc2-5E*, *esc2-5E-SLD2m* led to stronger MMS sensitivity and more severe growth defects when combining with *mms4Δ* (Fig. 5, *B* and *C*). The simplest interpretation of these genetic data is that though both the Esc2-MR domain and the SLD2 affect STR sumoylation, they also have separate roles.

### Esc2’s SUMO-interaction motif does not contribute to STR sumoylation or functions

Given the effects of Esc2 in STR sumoylation and functions, we examined another feature of Esc2 related to SUMO. Esc2 was reported to contain a SUMO-interaction motif (SIM) that is necessary and sufficient for binding SUMO (Fig. 2*A*) (28). Mutation of two key residues in SIM (V120A/V121A) disrupted the Esc2–SUMO interaction in yeast two-hybrid assay (28). Esc2’s SIM was shown to be important for transcriptional silencing (28). We generated the *esc2-SIMm* (V120A, V121A) allele and showed that it did not affect protein expression (Fig. S4). Unlike Esc2-MR and SLD2 mutants, *esc2-SIMm* supported WT level of STR sumoylation (Fig. S6*A*). In addition, *esc2-SIMm* did not sensitize *mms4Δ* for growth or for genotoxin survival (Fig. S6*B*). Thus, Esc2’s SIM neither contributes to STR sumoylation nor STR-mediated HJ removal.

## Discussion

STR plays important roles in the processing of HJs and in other HR steps. Sgs1 and its mammalian homolog BLM are both regulated by sumoylation; however, the underlying mechanisms are largely unclear (13, 14, 15, 19). Here, we provide biochemical and genetic evidence that multiple factors contribute to Sgs1 sumoylation. First, data derived from *in vitro* sumoylation assays suggest that Sgs1 binding to DNA stimulates its sumoylation. In principle, this could be achieved through conformational changes in favor of SUMO conjugation as seen for other substrates (29). This model provides one explanation for the observed dependency of Sgs1 sumoylation on HJ formation in cells (14). Second, we found that HJ-based stimulation of Sgs1 sumoylation is further enhanced by the Mms21–Smc5 SUMO E3 or the Esc2 scaffold protein, both of which have DNA-binding ability. While the DNA-binding sites of Mms21–Smc5 are unavailable, that of Esc2 has been mapped to its MR (20, 21). Third, using two Esc2-MR variants (Esc2-5E and -MRΔ), we provided evidence that the Esc2-MR domain has a previously unappreciated role in promoting Sgs1 sumoylation that is independent of its DNA-binding activity. Given that Esc2-5E disrupted both DNA binding and the stimulatory effect of Sgs1 sumoylation, the basic residues mutated in this allele likely contribute to both activities. Fourth, as Esc2-5E remained the association with the SUMO E2, the role of Esc2-MR in Sgs1 sumoylation is unlikely *via* directly mediating the Esc2–E2 binding. This feature distinguished the role of Esc2-MR from that of Esc2-SLD2 domain, which promotes Sgs1 sumoylation *via* binding to the SUMO E2 (18). Collectively, our *in vitro* data suggest a model wherein Sgs1 binding to DNA primes for its sumoylation while Esc2 uses two distinct domains to promote SUMO conjugation.

Our cellular results are consistent with *in vitro* biochemical data to support the roles of Esc2-MR domain in Sgs1 sumoylation. *In vivo* data further revealed a role of this domain in the sumoylation of Sgs1 partner complex Top3–Rmi1. Genetic analyses provide evidence suggesting that the Esc2-MR domain promotes STR-based functions. When using *mms4* synthetic interaction as a genetic readout for perturbing STR functions, *esc2-MRΔ* exhibited a stronger phenotype than *esc2-5E*. This may reflect a stronger defect associating with domain deletion than with point mutations of the domain. Interestingly, despite their similar impairment of STR sumoylation, *esc2-5E* was more potent in sensitizing *mms4* than *esc2-SLD2m* (Fig. 5*C*). The simplest interpretation is that unlike *esc2-SLDm*, *esc2-5E* also disrupted Esc2–DNA binding and possible other functions. As the combined mutation *esc2-5E-SLD2m* showed worsening phenotype than *esc2-5E* or -*SLD2m* single mutant, it is likely that Esc2-MR and Esc2-SLD2 have separate roles in cells. Esc2-MR has been implicated in the regulation of two other HR factors, namely the antirecombinase Srs2 and the Mms4-Mus81 nuclease, whereas Esc2-SLD2 is involved in the sumoylation of substrates including the replication polymerases and helicases (18, 20, 21). Whether the two Esc2 domains collaborate or act independently for these previously noted functions will be interesting to address in the future. In addition, future research should address whether and how Sgs1 sumoylation affects its roles in multiple HR steps such as DNA end resection or D-loop disassembly and how this modification influences its helicase activity. We also examined the Esc2-SIM sequence that has been implicated in transcriptional silencing (28) and concluded its lack of involvement in STR sumoylation and HJ removal. Thus, our work, in conjunction with previous studies, reveals multifunctional nature of the Esc2 scaffold protein. These findings can guide the investigation of its mammalian homologs such as the Nip45 protein involved in genome maintenance in animals (30).

In summary, our combination of *in vitro* sumoylation and cell-based data provided evidence for the direct role of DNA in stimulation of Sgs1 sumoylation and an unexpected function of the Esc2-MR in sumoylation. These findings deepen our understanding of the mechanism underlying the sumoylation regulation of STR genome maintenance complex.

## Experimental procedures

### Yeast strains and genetics procedures

Yeast strains are derivatives of W1588-4C, a *RAD5* derivative of W303 *(MATa ade2-1 can1-100 ura3-1 his3-11,15 leu2-3112 trp1-1 rad5-535*). Mutations were introduced using a standard one-step integration PCR-based method. Correct tagging and mutations were verified by sequencing. Standard procedures were used for media preparation, cell growth, epitope tagging at endogenous loci, mutant generation, and spot assays. At least two strains per genotype were used in each experiment, and only one is listed in the Table S1.

### DNA substrates

The HJ and dsDNA substrates were made by annealing the 80-mer oligos listed in Table S2. The annealed substrates were then gel purified and concentrated in TE buffer (10 mM Tris-Cl, 1 mM EDTA, pH 8.0). For DNA mobility shift assay substrates, one of these 80-mer oligo was P^32^ labeled, and the substrates were made following the same procedure.

### Protein expression and purification

The expression and purification of most recombinant proteins used for *in vitro* analysis were performed following previously published procedures. Specifically, Smt3, Smt3-D68R, Aos1-Uba2 (SUMO E1), Ubc9 (SUMO E2), the Mms21 (SUMO E3)–Smc5 complex, V5-Top3/GST-Rmi1, and Esc2 and Esc2-SLD2m were expressed and purified from *E. coli* (18, 20, 31, 32, 33). Flag-Sgs1 was expressed and purified from High Five insect cells (9). STR complex was assembled *in vitro* using 1:1 mol ratio of purified Sgs1 and V5-Top3–GST-Rmi1 complex. The coding sequence of Esc2-MRΔ truncating residues 154 to 198 and Esc2-5E mutant (K179E, K182E, K183E, K197E, and R198E) were synthesized and inserted into the pET24a expression vector (Gene Universal). The expression and purification of the two Esc2 mutants followed published procedures (18, 20).

### *In vitro* sumoylation assays

The *in vitro* sumoylation assays for Sgs1 was performed as described (18), except for a few modifications. The basal sumoylation reactions in Figure 1 were carried out by first incubating 20 nM STR complex with 50 nM Aos1-Uba2 (E1), 280 nM Ubc9 (E2), and 2.2 μM SUMO-D68R. SUMO-D68R did not affect sumoylation reaction efficiency but allows the detection of Esc2-based stimulation of Sgs1 sumoylation as shown previously. For reactions containing the SUMO E3, 25 nM Mms21/Smc5 was added. For reactions that contained DNA, 40 nM HJ substrate as described previously (34) or 40 nM 80-mer dsDNA was added. The sumoylation reaction buffer R contains 45 mM Hepes-Na (pH 7.0), 5 mM MgCl_2_, 65 mM KCl, and 0.1 mM DTT. The sumoylation reaction was initiated by adding 5 mM ATP and incubating at 30 °C. Samples were taken at indicated time points and mixed with sample loading buffer.

To increase the stringency of reaction conditions, sumoylation reactions shown in Figure 2, Figure 3, Figure 4 contained 30 nM STR complex, 2.2 μM SUMO-D68R, 50 nM Aos1-Uba2 (E1), 280 nM Ubc9 (E2), and 40 nM Mms21–Smc5 complex (E3). For reactions containing Esc2, 300 nM Esc2 WT or variants were added. For reactions that contained DNA, 40 nM HJ substrate or 40 nM 80-mer dsDNA was added. The reaction buffer R^high salt^ contains 45 mM Hepes-Na (pH 7.0), 5 mM MgCl_2_, 80 mM KCl, and 0.1 mM DTT. The sumoylation reaction was initiated and performed as described previously. All samples were analyzed by either 4 to 20% gradient or 7.5% SDS-PAGE and immunoblotting using anti-FLAG antibody (Sigma) recognizing the FLAG tag on Sgs1 or using anti-V5 antibody (Rockland) recognizing the V5 tag on Top3. All sumoylation experiments were repeated twice, and sumoylated Sgs1 was quantified using ImageQuant TL (GE Healthcare).

### DNA mobility shift assay

For STR complex and Mms21/Smc5 DNA binding, STR (5–40 nM) or Mms21/Smc5 (5–40 nM) was incubated with the aforementioned radiolabeled HJ or dsDNA (5 nM) at 30 °C for 10 min in 10 μl of buffer D (35 mM Tris–HCl, pH 7.5, 1 mM DTT, 100 μg/ml bovine serum albumin, 5 mM MgCl_2_, and 130 mM KCl). The reaction mixtures were mixed with DNA loading buffer, and the resulting mixture was then resolved in 6.5% polyacrylamide gels in TAE buffer (40 mM Tris–acetate and 1 mM EDTA). For Esc2 DNA binding, 5 to 80 nM Esc2 WT or its mutants (Esc2-5E or Esc2-MRΔ) was incubated with the HJ or dsDNA (5 nM) at 30 °C for 10 min in 10 μl of buffer D. The reaction mixtures were mixed with DNA loading buffer, and the resulting mixture was then resolved in 7% polyacrylamide gels in TAE buffer.

### *In vitro* pull-down assay

GST pull-down assay was performed following our published procedure (18). Briefly, GST or GST-tagged Esc2 or its variants at a final concentration of 2.2 μM was incubated with 3.6 μM (final concentration) of Ubc9 in 30 μl of T buffer (25 mM Tris-Cl, pH 7.4, 10% glycerol, 0.5 mM EDTA, 0.01% Igepal, 1 mM DTT) supplemented with 80 mM KCl for 30 min at 4 °C. The protein mixture was then incubated with 10 μl of Glutathione Sepharose 4B resin (GE Healthcare) for 30 min at 4 °C. After washing the resin four times with 200 μl of T buffer with 80 mM KCl, bound proteins were eluted with 20 μl of sample loading buffer. Ten percent of the supernatant (S) and eluate (E) fractions and 2% of the wash (W) fraction were analyzed by 4% to 20% SDS-PAGE.

### Protein sumoylation detection in cell extract

Detection of protein sumoylation was conducted as previously described (18). Briefly, exponentially growing cells containing His8-tagged SUMO were treated with 0.03% MMS for 2 h and 1 × 10^9^ cells were collected. Protein extracts prepared by 55% trichloroacetic acid were dissolved in buffer A (6 M guanidine HCl, 100 mM sodium phosphate, 10 mM Tris–HCl adjust to pH 8.0) and incubated overnight with nickel-nitrilotriacetic acid resin after the addition of 0.05% Tween 20 and 4.4 nM imidazole. Resins were washed twice with buffer A containing 0.05% Tween 20 and four times with buffer C (8 M urea, 100 mM sodium phosphate, 10 mM Tris–HCl adjust to pH 6.3) containing 0.05% Tween 20. The bound proteins were eluted by HU buffer (8 M urea, 200 mM Tris–HCl at pH 6.8, 1 mM EDTA, 5% SDS, 0.1% bromophenol blue, 1.5% DTT, 200 mM imidazole), then examined by SDS-PAGE and immunoblotting analyses. Ponceau S stain was used to ensure equal loading.

### Statistical analysis

Statistical analysis was performed using unpaired Student’s *t* test. Data are expressed as mean ± SD. All experiments were repeated at least two times. Values of *p* < 0.05 were considered significant.

## Data availability

The data used and/or analyzed in the current study are available from the corresponding author on reasonable request.

## Supporting information

This article contains supporting information (18).

## Conflict of interest

The authors declare that they have no conflicts of interest with the contents of this article.
