# Supplementary material for: Multifaceted regulation of the sumoylation of the Sgs1 DNA helicase
Source: J Biol Chem. 2022 May 30;298(7):102092. doi: 10.1016/j.jbc.2022.102092 (PMC9243176; doi:10.1016/j.jbc.2022.102092)
Supplement: Supporting Information [file mmc1.docx]

**­­­**

**Supporting Information**

**Multi-faceted regulation of the sumoylation of the Sgs1 DNA helicase**

Shibai Li^1, 4^, Ashley Mutchler^2, 4^, Xinji Zhu^3^, Stephen So^3^, John Epps^3^, Danying Guan^1^,

Xiaolan Zhao^1^ *, and Xiaoyu Xue^2, 3,^ *

**Supporting Tables**

**Table S1. Yeast strains used in this study**

| **Name** | **Genotype** | **Source** |
| --- | --- | --- |
| X7559-8A | *SGS1-9myc::KAN 8His-SMT3::TRP1 ESC2-10FLAG::KAN* | Li et al. 2021 |
| X7704-5A | *SGS1-9myc::KAN 8His-SMT3::TRP1 esc2Δ::KAN* | Li et al. 2021 |
| X7782-2D | *SGS1-9myc::KAN 8His-SMT3::TRP1 esc2-∆154-198aa-10FLAG::KAN* | This study |
| X8391-8C | *SGS1-9myc::KAN 8His-SMT3::TRP1 esc2*-*5E-10FLAG::KAN* | This study |
| X7555-5B | *TOP3-TAP::HIS3 8His-SMT3::TRP1 ESC2-10FLAG::KAN* | Li et al. 2021 |
| X7705-1C | *TOP3-TAP::HIS3 8His-SMT3::TRP1 esc2Δ::KAN* | Li et al. 2021 |
| X7783-2B | *TOP3-TAP::HIS3 8His-SMT3::TRP1 esc2-∆154-198aa-10FLAG::KAN* | This study |
| X8408-7B | *TOP3-TAP::HIS3 8His-SMT3::TRP1 esc2*-*5E-10FLAG::KAN* | This study |
| X7556-13B | *RMI1-TAP::HIS3 8His-SMT3::TRP1 ESC2-10FLAG::KAN* | Li et al. 2021 |
| X7706-11B | *RMI1-TAP::HIS3 8His-SMT3::TRP1 esc2Δ::KAN* | Li et al. 2021 |
| X7784-3D | *RMI1-TAP::HIS3 8His-SMT3::TRP1 esc2-∆154-198aa-10FLAG::KAN* | This study |
| X8392-1C | *RMI1-TAP::HIS3 8His-SMT3::TRP1 esc2*-*5E-10FLAG::KAN* | This study |
| X7556-9D | *ESC2-10FLAG::KAN* | Li et al. 2021 |
| X8020-2-11C | *esc2Δ::KAN* | This study |
| X8022-2-10A | *esc2-∆154-198aa-10FLAG::KAN* | This study |
| X8423-5D | *esc2*-*5E-10FLAG::KAN* | This study |
| X8021-2-7D | *esc2-SLD2m-10FLAG::KAN* | This study |
| X8448-12D | *esc2-SLD2m-5E-10FLAG::KAN* | This study |
| X8423-4C | *mms4Δ::KAN* | This study |
| X8019-12A | *mms4Δ::KAN ESC2-10FLAG::KAN* | This study |
| X8020-9A | *mms4Δ::KAN esc2Δ::KAN* | This study |
| X8022-8B | *mms4Δ::KAN esc2-∆154-198aa-10FLAG::KAN* | This study |
| X8423-4B | *mms4Δ::KAN esc2*-*5E-10FLAG::KAN* | This study |
| X8021-14A | *mms4Δ::KAN esc2-SLD2m-10FLAG::KAN* | This study |
| X8448-12C | *mms4Δ::KAN esc2-SLD2m-5E-10FLAG::KAN* | This study |
| T2199-8 | *esc2-sim-10FLAG::KAN* | This study |
| X8806-19A | *mms4Δ::KAN esc2-sim-10FLAG::KAN* | This study |
| X8614-1A | *SGS1-9myc::KAN 8His-SMT3::TRP1 esc2-sim-10FLAG::KAN* | This study |
| X8603-6C | *TOP3-TAP::HIS3 8His-SMT3::TRP1 esc2-sim-10FLAG::KAN* | This study |
| X8602-1B | *RMI1-TAP::HIS3 8His-SMT3::TRP1 esc2-sim-10FLAG::KAN* | This study |
| X8890-16B | *mms4Δ::KAN esc2-5E-10FLAG::KAN rad51∆::LEU2* | This study |
| X8888-3B | *mms4Δ::KAN esc2∆::KAN rad51∆::LEU2* | This study |

**Table S2. DNA Substrates used in this study.**

| Substrate | Oligo Name | Length | Sequence |
| --- | --- | --- | --- |
| HJ | H3 | 80 | 5’-TTGATAAGAGGTCATTTGAATTCATGGCTTAGAGC TTAATTGCTGAATCTGGTGCTGGGATCCAACATGTTTTAAATATG-3’ |
|  | H5 | 80 | 5’-CATATTTAAAACATGTTGGATCCCAGCACCAGATT CAGCATACGTTACCGATCGTACGTTCGATGCTGGCTACTGCTAGC-3’ |
|  | H7 | 80 | 5’-GCTAGCAGTAGCCAGCATCGAACGTACGATCGGT AACGTAGTCGATTATCGAGATCAAGCTAGCATAGCCATAGCGCGAC-3’ |
|  | H8 | 80 | 5’-GTCGCGCTATGGCTATGCTAGCTTGATCTCGATAA TCGACATTAAGCTCTAAGCCATGAATTCAAATGACCTCTTATCAA-3’ |
| dsDNA | H3 | 80 | 5’-TTGATAAGAGGTCATTTGAATTCATGGCTTAGAGC TTAATTGCTGAATCTGGTGCTGGGATCCAACATGTTTTAAATATG-3’ |
|  | H4 | 80 | 5’- CATATTTAAAACATGTTGGATCCCAGCACCAGAT TCAGCAATTAAGCTCTAAGCCATGAATTCAAATGACCTCTTATCAA-3’ |

**Supporting Figures**

**
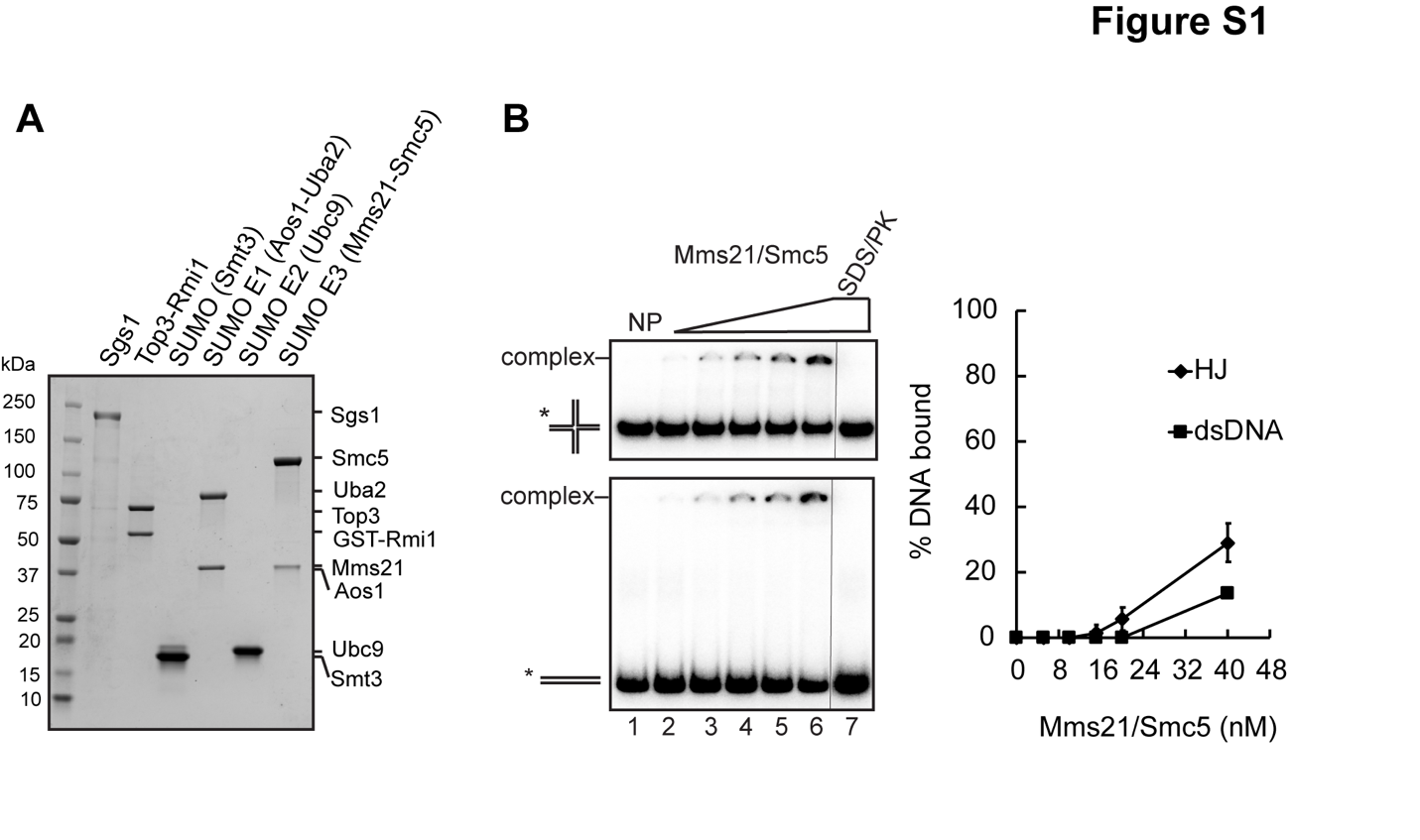
**

**Figure S1. DNA mobility shift assay of the Mms21/Smc5 SUMO E3 complex.**

*A*, purified proteins used in the *in vitro* studies. Proteins were analyzed by SDS-PAGE and stained with Coomassie blue.

*B*, DNA mobility shift assay for the Mms21/Smc5 SUMO E3 complex. The assay was performed as in Figure 1B. The Mms21/Smc5 complex (5-40 nM) was incubated with 5 nM of HJ-DNA or dsDNA. The results were quantified and plotted as mean ± SD (n=3 technical replicates).

**
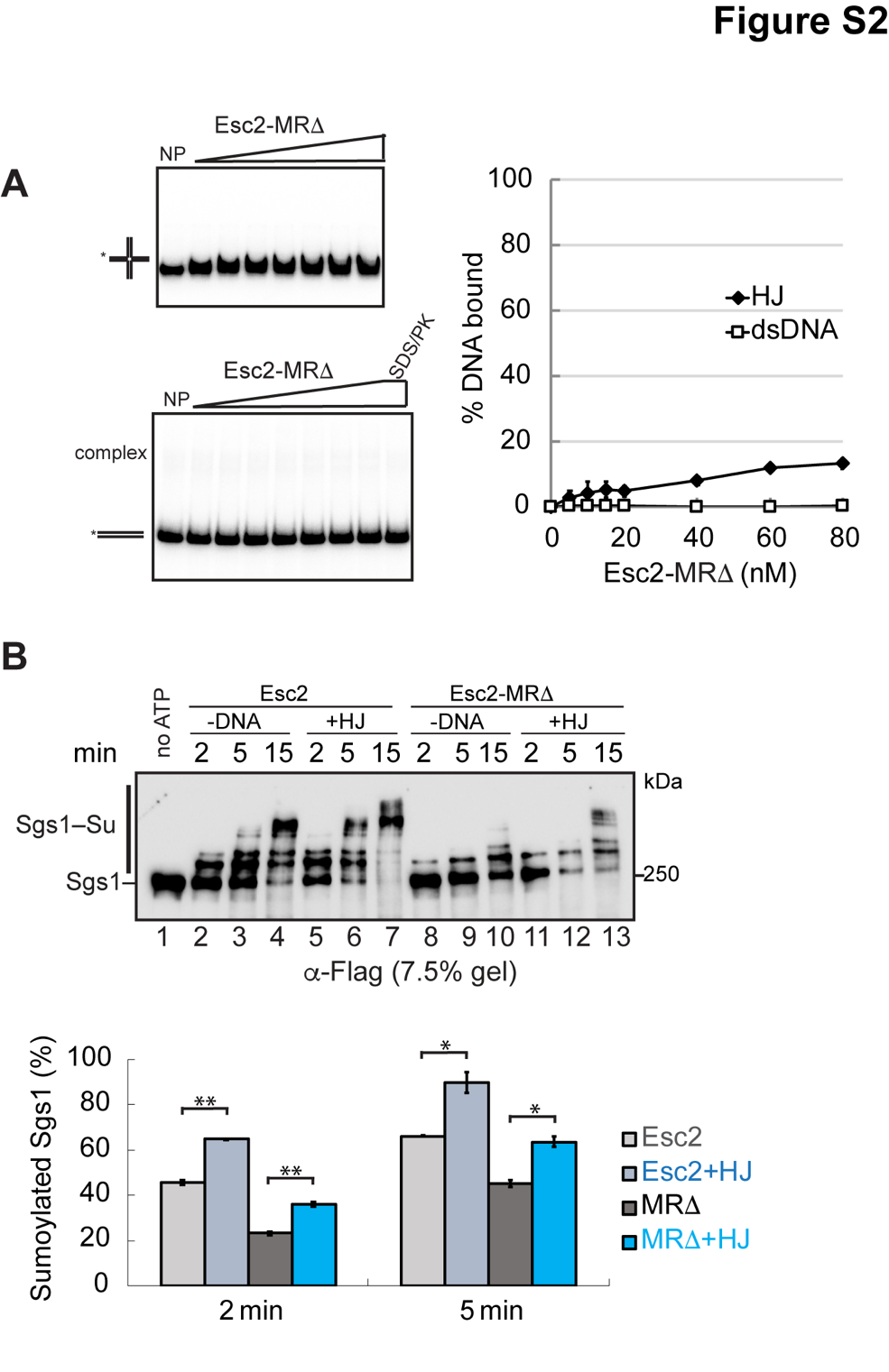
**

**Figure S2. Esc2-MR∆ abolished DNA binding and reduced the stimulatory effect on Sgs1 sumoylation.**

*A*, DNA mobility shift assay showed that Esc2-MR∆ failed to binds to HJ-DNA or dsDNA. The mean ± SD from at least three independent experiments were plotted.

*B*, The effect of Esc2-MR∆ on Sgs1 sumoylation. Assays were performed and data are presented as in Figure 3B. The quantified percentage of sumoylated Sgs1 showed mean ± SD (n=2 technical replicates). *, p<0.05; **, p<0.01; ***, p<0.001.

**
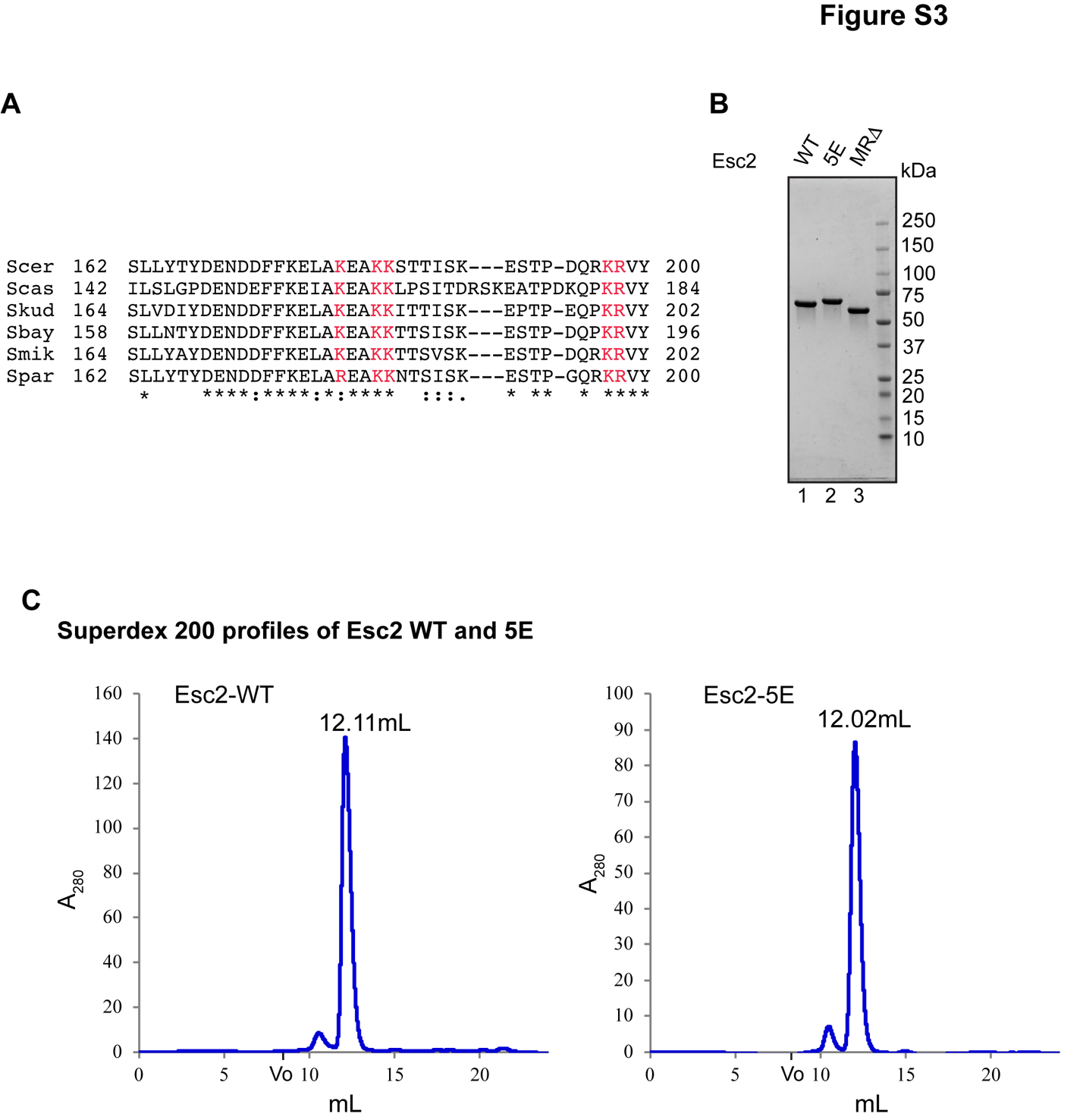
**

**Figure S3. Purification of wild-type and mutant Esc2 proteins**

*A*, alignment of a mid-region of the Esc2 sequence with those from its yeast homologs. The conserved basic residues changed to glutamate in the Esc2-5E mutant are colored red. *Scer: Saccharomyces cerevisiae*, *Scas*: *Saccharomyces castellii*, *Sbay*: *Saccharomyces bayanus*, *Spar*: *Saccharomyces paradoxus*, *Skud*: *Saccharomyces kudriavzevii,* *Smik, Saccharomyces mikatae.*

*B*, purified Esc2, Esc2-5E and Esc2-MR∆ proteins were analyzed by SDS-PAGE and stained with Coomassie blue.

*C*, the purified Esc2 and Esc2-5E were analyzed by Superdex 200 gel filtration, and the elution profiles were shown. The void volume of the Superdex 200 column (Vo) was indicated.

**
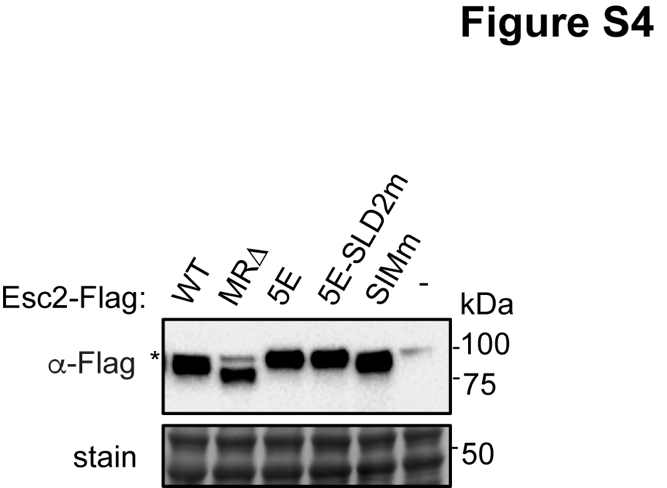
**

**Figure S4. Esc2 mutant protein levels.**

Protein extracts from cells with indicated genotype were examined by immunoblotting. Wild-type control cells containing untagged Esc2 exhibited a cross-reactive band (*).

**Figure S5. The negative genetic interaction between *esc2* and *mms4∆* mutants is suppressed by *rad51∆*.** Cells were spotted in 10-fold serial dilutions and grown for 2 days at 30oC.

**
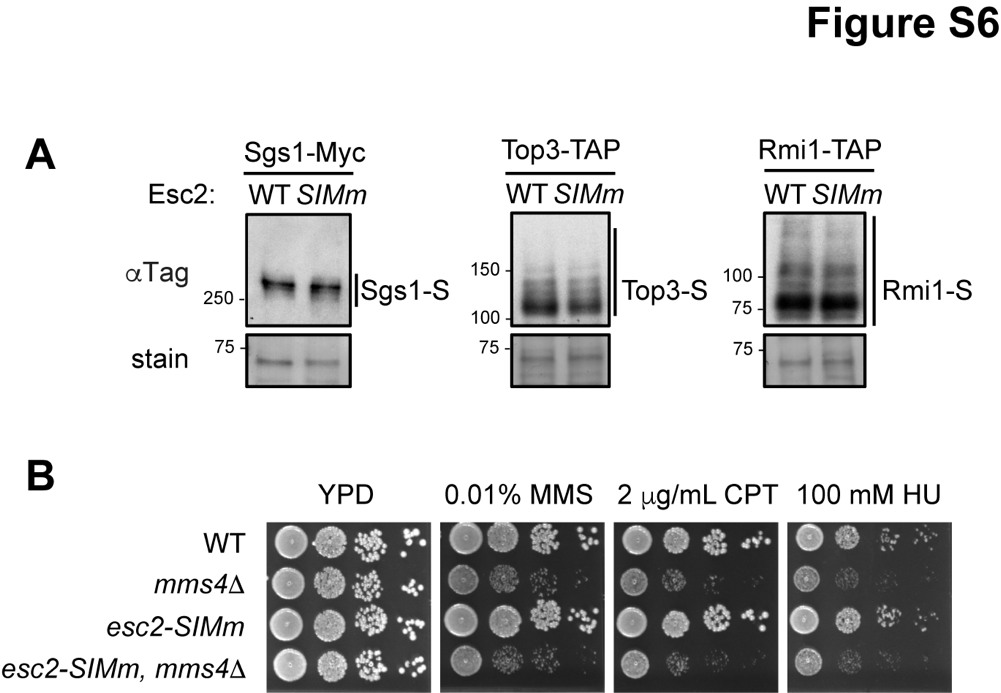
**

**Figure S6. *esc2-SIMm* mutant phenotype**

*A*, STR sumoylation was examined in *esc2-SIMm* cells. Experiments were performed and data are present as Figure 5A.

*B*, *esc2-SIMm* mutant did not worsen genotoxic sensitivity of *mms4∆* cells. Cells were spotted in 10-fold serial dilutions and grown for 2 days at 30^o^C.
